# Supplementary material for: The role of platelets in mediating a response to human influenza infection
Source: Nat Commun. 2019 Apr 16;10:1780. doi: 10.1038/s41467-019-09607-x (PMC6467905; doi:10.1038/s41467-019-09607-x)
Supplement: Supplementary file 1 — Supplementary Information [file 41467_2019_9607_MOESM1_ESM.pdf]

The Role of Platelets in Mediating a Response to Human Influenza Infection

Koupenova, et all.

Supplementary Figures

Supplementary Figure 1: Additional images of platelets found in the blood of influenza-infected patients.

Supplementary Figure 2: Released DNA in blood from influenza-infected patients is mostly of neutrophil origin.

Supplementary Figure 3: Cell free DNA in plasma of influenza-infected patients.

Supplementary Figure 4: Blood neutrophil lacking CD66b neutrophil marker in influenza-infected patient.

Supplementary Figure 5: Brake down of the images that formed the merged confocal microscopy overlay in Figure 1h (top panel).

Supplementary Figure 6: Influenza particles are found in/with platelets and caught in released-DNA-platelet aggregates.

Supplementary Figure 7: TEM images of platelets incubated with influenza.

Supplementary Figure 8: TEM images of platelets isolated from blood of influenza infected patients.

Supplementary Figure 9: C3 levels as a function of aspirin intake; TLR7 stimulation and presence in platelets.

Supplementary Figure 10: Platelet TLR7 does not always co-localize with lysosomal markers and is predominantly intracellular.

Supplementary Figure 11: Influenza particles are not always found in the same platelets that express TLR7.

Supplementary Figure 12: TLR7 activation of platelets leads to platelet cross-communication.

Supplementary Figure 13: Ex vivo treatment of human blood with a TLR7 agonist leads to DNA release from neutrophils.

Supplementary Figure 14: Purity of neutrophils utilized throughout our study.

Supplementary Figure 15: Supplementary Figure 9: Levels of CCL5 (RANTES) in plasma of influenza-infected patients.

Supplementary Figure 16: Neutrophil-DNA release in mouse blood after 4h of in vivo TLR7 stimulation.

Supplementary Figure 17: Neutrophil-DNA release in murine blood after 24h of in vivo infection with influenza.

Supplementary Figure 18: Presence of influenza in murine blood post-infection.

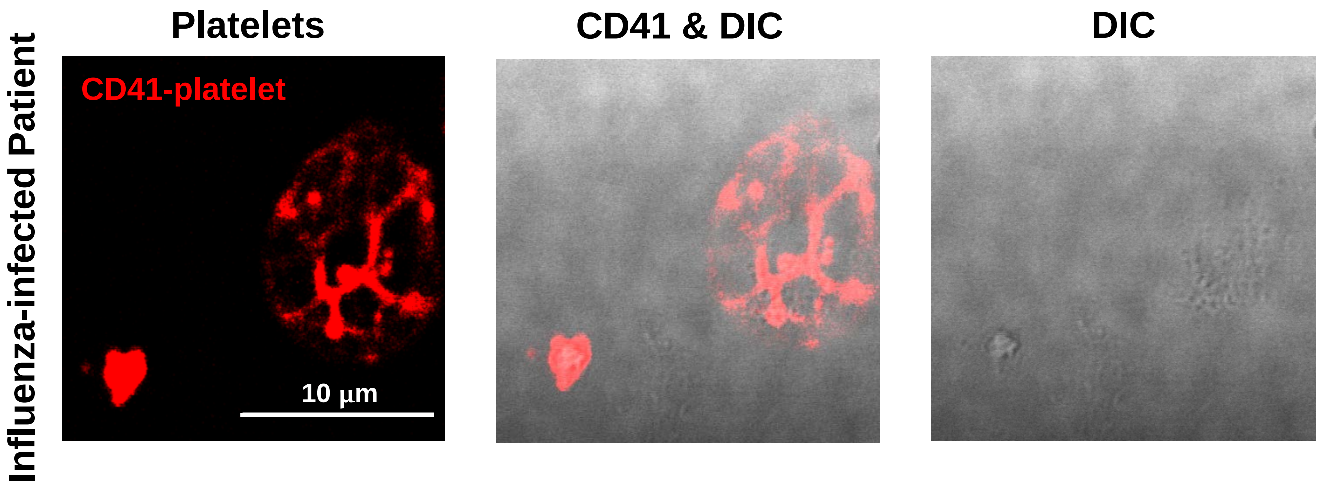

Supplementary Figure 1: Additional images of platelets found in the blood of influenza-infected patients. Fifty µL of intravenous blood drawn from influenza positive patients or healthy donors was fixed (red blood cells were lysed) and samples were stained with respective antibodies. A platelet with flattened satellite-like morphology visualized by CD41-APC (red). Merged image of CD41 and differential interference contrast (DIC) shows that this is a singular platelet and not platelet aggregates. Aggregates of platelets by DIC can be seen in Supplementary Figure 10b**.**

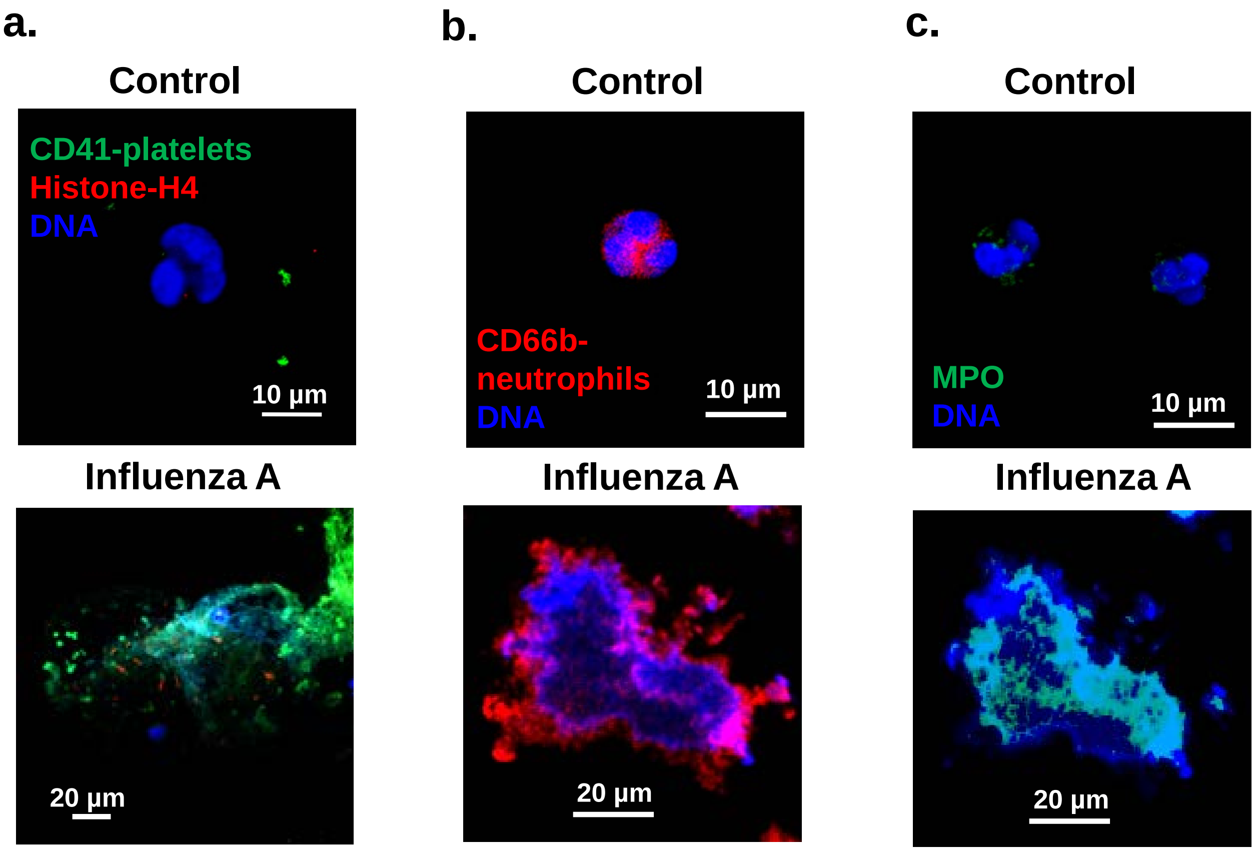

Supplementary Figure 2: Released DNA in blood from influenza-infected patients is mostly of neutrophil origin. Blood from influenza-infected patients was fixed after intravenous collection and stained as described in the Methods. In all cases DNA was assessed by DAPI. **a.** Association of platelets with released-DNA found in human blood from influenza-infected donors. DNA was covered with platelets and histones (red). **b-c**. Released DNA is of neutrophil origin assessed by **b.** the neutrophil marker CD66b and **c.** the granule enzyme Myeloperoxidase (MPO). Representative images of 5 different patients infected with influenza are shown here. Of note: Blood from influenza-infected patients and controls was not permeabilized. Since formaldehyde can cause certain levels of permeabilization as a function of cross-linking, positive staining in control samples for H4 and MPO do not necessarily indicate activation.

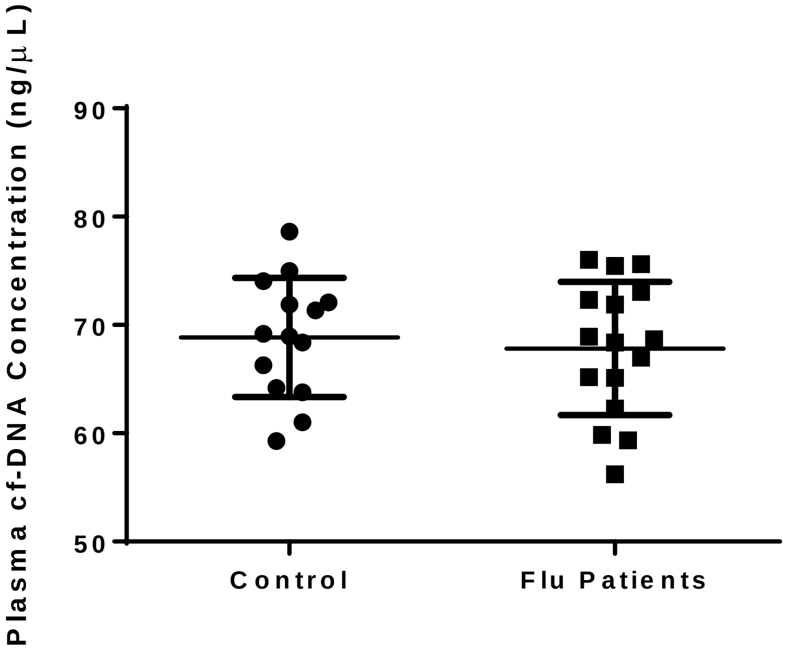

Supplementary Figure 3: Cell free DNA in plasma of influenza-infected patients. Cell free DNA was assessed by PicoGreen®·as described in the Methods utilizing citrated plasma from the patients and compared to control. No significance was assessed by Mann Whitney test. Data is average ± SD of n=14 uninfected controls and n=16 infected patients.

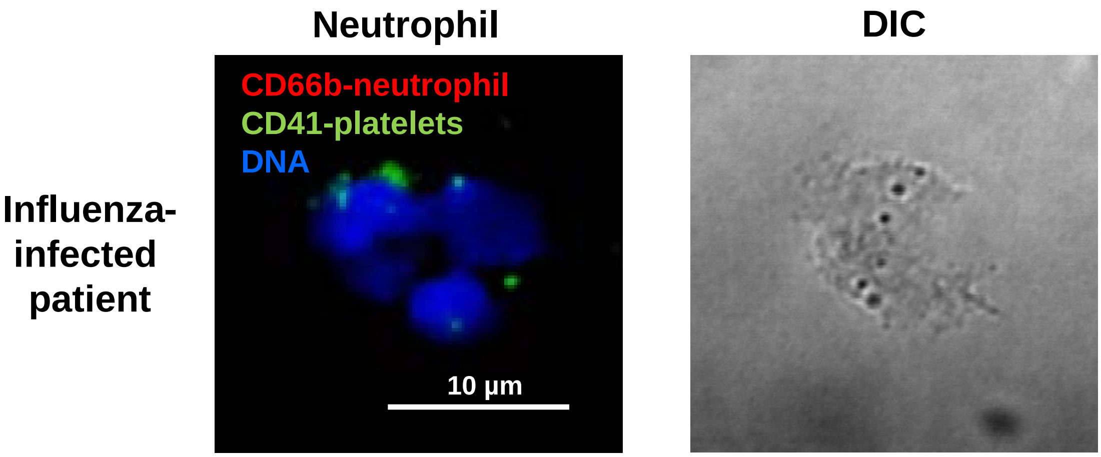

Supplementary Figure 4: Blood neutrophil lacking CD66b neutrophil marker in influenza-infected patient. Blood from influenza-infected patients was fixed after intravenous collection and stained with antibodies for the platelet marker CD41, neutrophil marker CD66b, and for DNA with DAPI. This neutrophil does not appear to be positive for CD66b and it is forming aggregates with released DNA as the membrane is lost, suggesting that certain released DNA in blood cannot be efficiently tracked back to neutrophils.

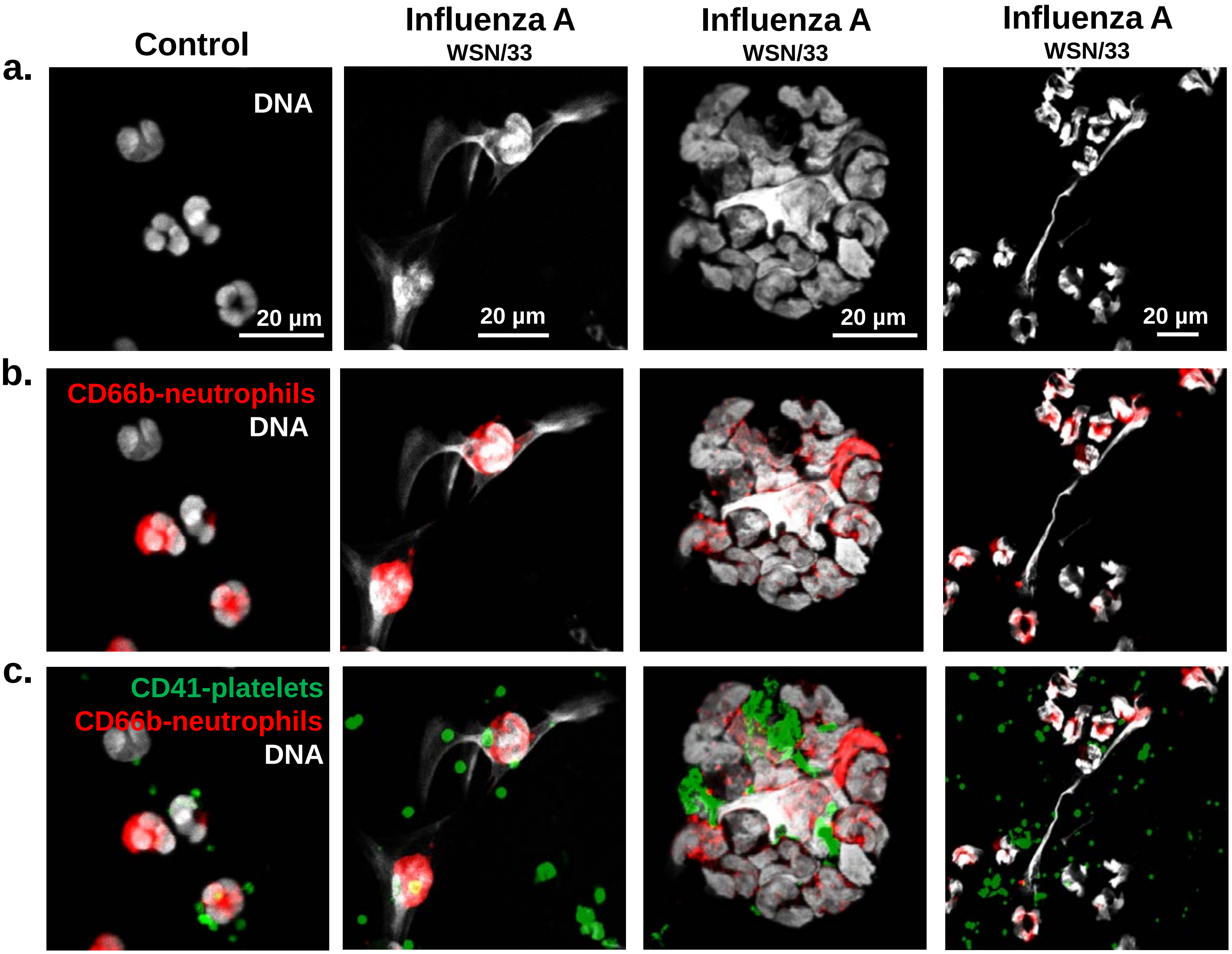

Supplementary Figure 5: Breakdown of the images that formed the merged confocal microscopy overlay in Figure 1h (top panel). Blood from human donors was treated for 30 min with sucrose-purified infectious influenza (WSN/33) at constant rotation and 37°C. Influenza was used at 1 pfu to 100 platelets. **a**. DNA fluorescence assessed by DAPI. **b**. neutrophil marker CD66b merged with the DNA image from a. **c**. Platelet CD41 overlayed with the image in b. Of note, in certain cases the DNA is not entirely covered with platelets, suggesting differences in kinetics of interaction and/or a physiological relationship that needs further in vivo characterization.

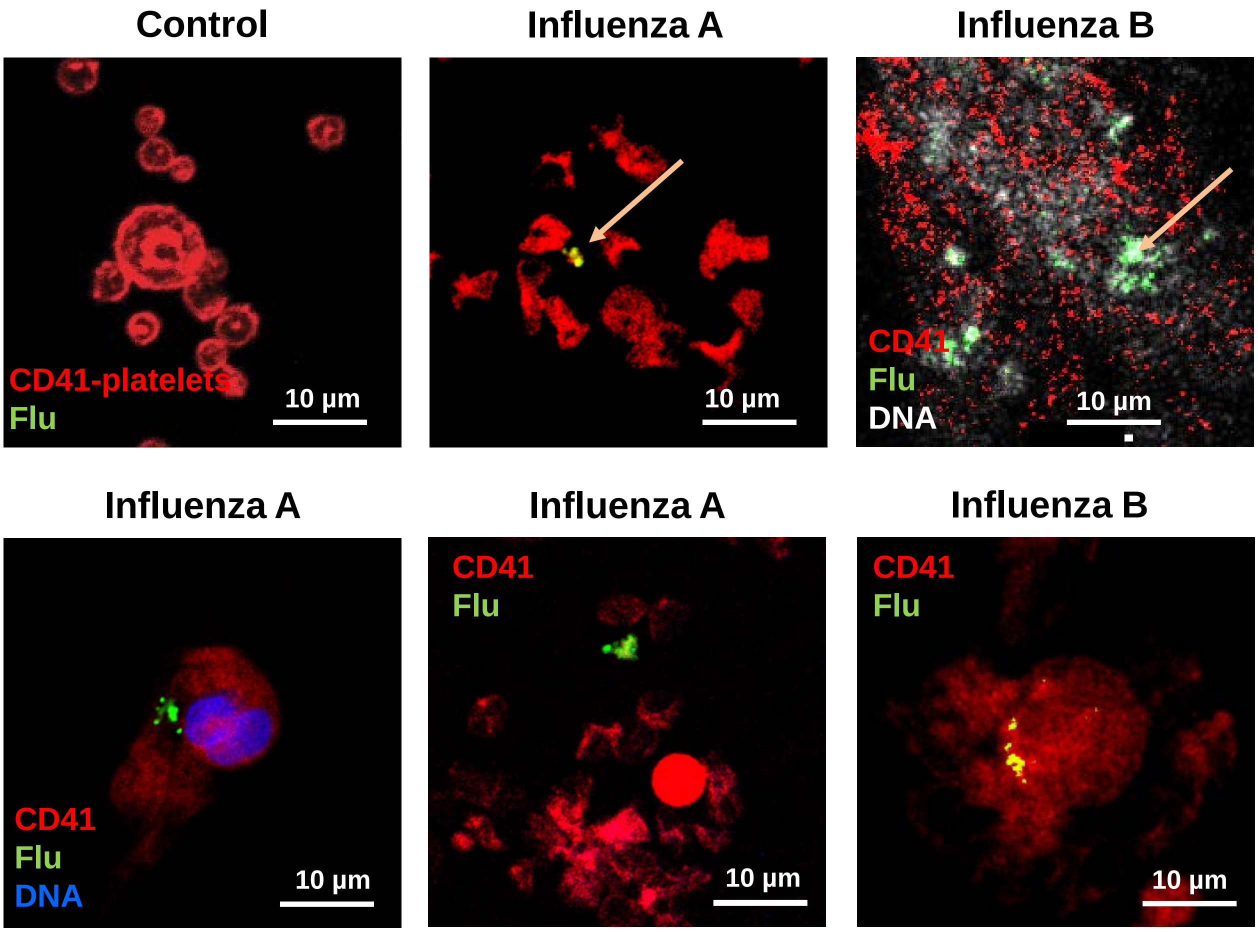

Supplementary Figure 6: Influenza particles are found in/with platelets and caught in released-DNA-platelet aggregates. Blood from influenza-infected patients was stained as described in Figure 1. Influenza particles predominantly found in platelets (or associated with platelets). Blood was stained with antibodies for the nuclear protein for influenza A (green) or influenza B (green, yellow=green and red combined) in platelets stained with CD41-APC (red); DNA from neutrophils (white). Images were resolved by confocal microscopy. Representative images are shown from n=5 patients.

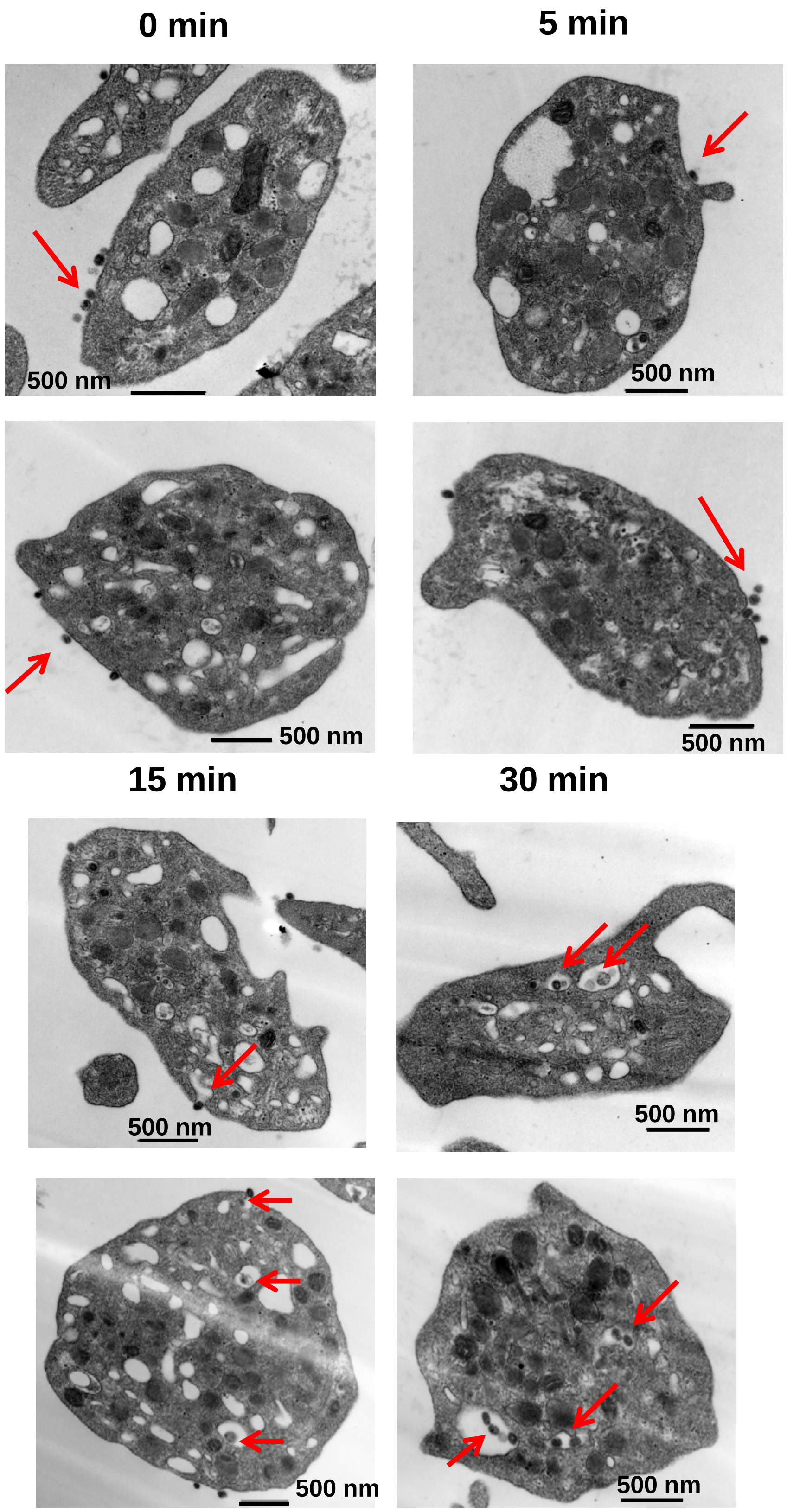

Supplementary Figure 7: TEM images of platelets incubated with influenza . Transmission electron micrographs of isolated human platelets incubated with WSN/33 influenza (1 pfu to 10 platelets), for the indicated time, at 1000 rpm (in PAP-8 aggregometer), 37°C. Platelets were fixed with EM-fixative for 10 min before processing. Arrows point to the viral particles.

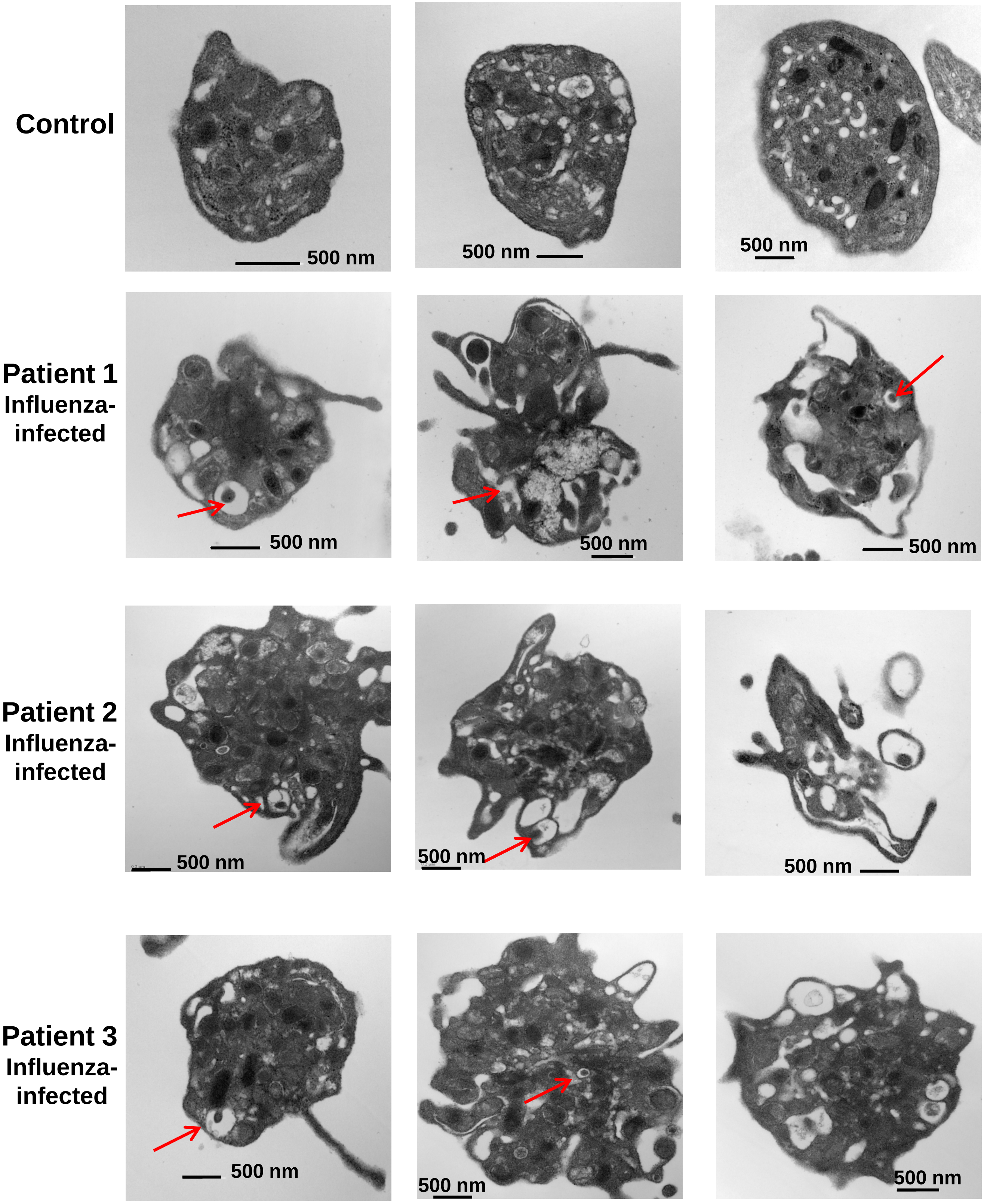

Supplementary Figure 8: TEM images of platelets isolated from blood of influenza infected patients. Transmission electron micrographs of isolated human platelets from non-infected (control) and influenza-infected patients. Platelets were stored in EM fixative for a few days before processing; arrows point to what could be viral particles based on morphology.

a.

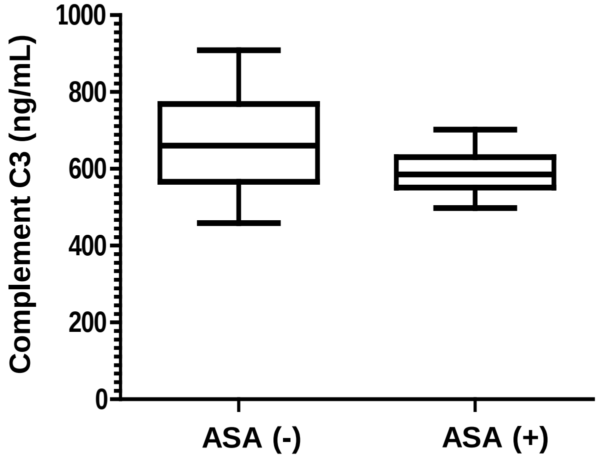

b.

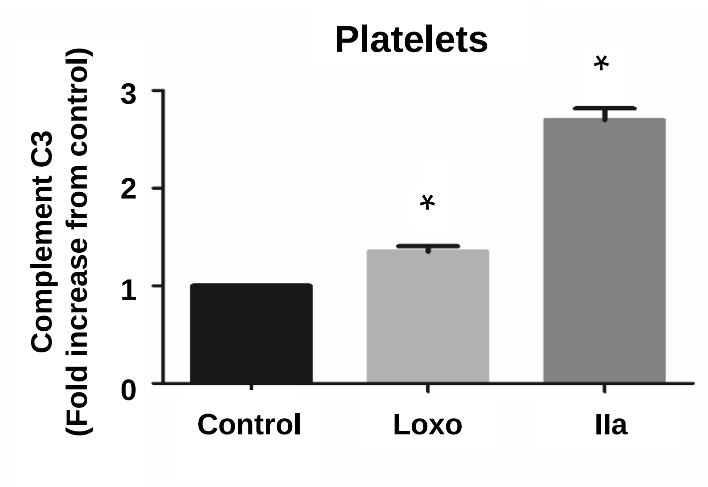

c.

C3 (ng/mL)	Control	Agonist	IRS661+ Agonist	IRS661
Flu	1265.35	1869.30	1498.89	1251.63
TLR7	1215.05	1861.79	1389.47	1214.50

Supplementary Figure 9: C3 levels as a function of aspirin intake; TLR7 stimulation and presence in platelets. a. C3 levels in the influenza-infected patients as a function of aspirin (ASA, acetylsalicylic acid) intake (n=8) and non-aspirin (n=10). The graphs in a. represent average ± SD; significance was assessed by Mann-Whitney U test. **b.** Washed human platelets were treated with TLR agonists [TLR7 (loxoribine, Loxo) – 1 mM; thrombin (IIa) - 0.05 U/mL] for 30 min and C3 was measured by ELISA. The graph represents the average fold change for each individual of n=7 (5F; 2M) ± SEM. Significance was measured by ANOVA followed by Bonferroni follow-up test with * p<0.001. **c.** Representative levels of C3 secretion from platelets of the same donor stimulated with either influenza or Loxo.

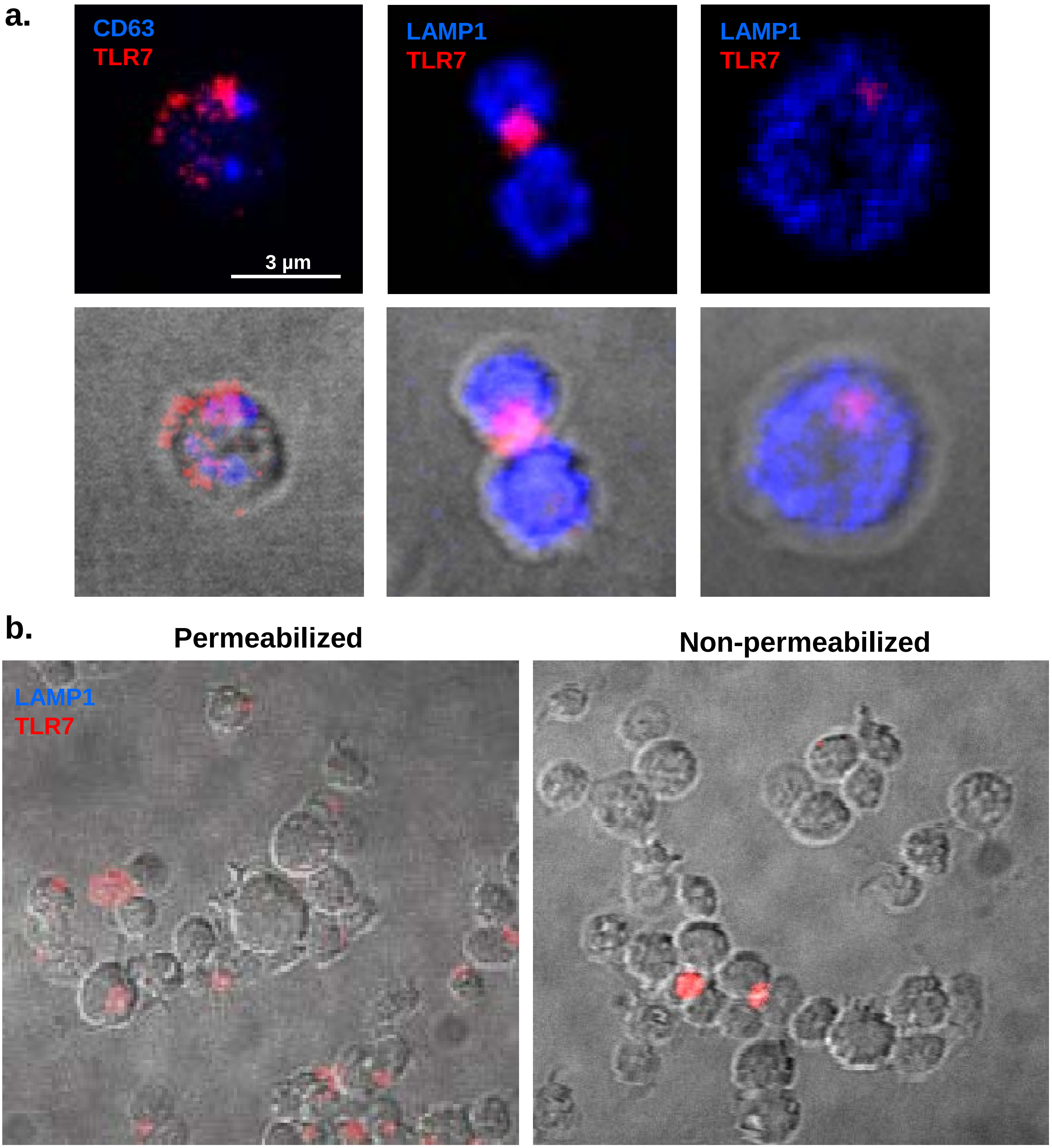

Supplementary Figure 10: Platelet TLR7 does not always co-localize with lysosomal markers and is predominantly intracellular. Platelets isolated from healthy human donors were incubated with WSN/33 influenza strain as in Supplementary Figure 2. Cells were permeabilized and stained with different antibodies. **a.** Representative confocal microscopy images of platelets from 3 different donors stained with TLR7-APC and with either of the lysosomal markers: CD63-BV421 (LAMP3) or LAMP1-DyLight405 (CD107a). There is a certain amount of TLR7 that does not colocalize with CD63, suggesting that TLR7 is not present in the lysosomes at all times. **b.** Confocal microscopy of permeabilized and non-permeabilized platelets stained with TLR7-APC, and LAMP1-DyLight405 (CD107a). Images were colocalized with DIC. LAMP1 was rarely found in certain donors. TLR7 is mostly intracellular, although in certain donors there is some surface expression (~5% of non-permeabilized platelets). Images are representative of n=5 donors (3F, 2M).

a.

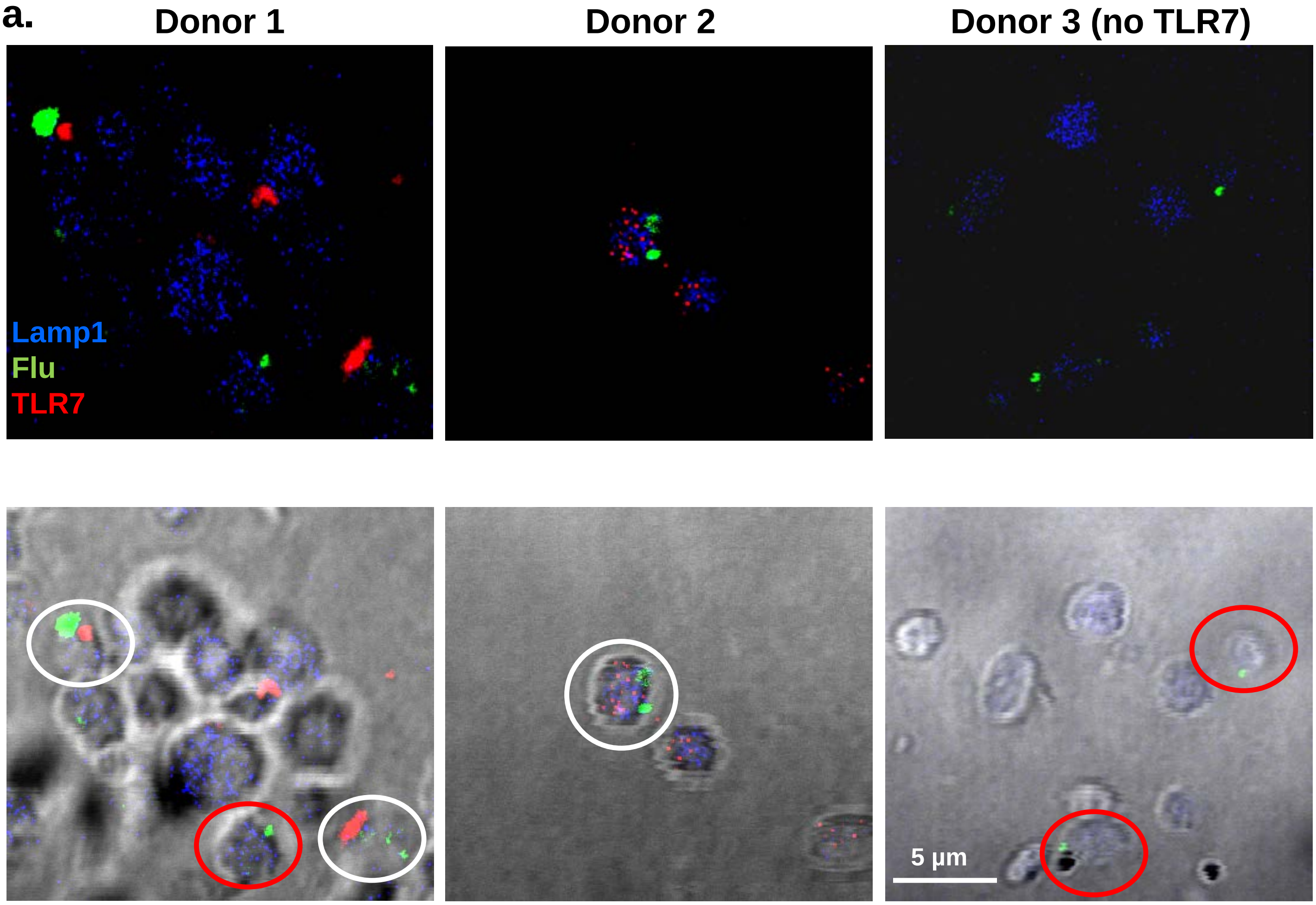

b.

dCT	Donor 1	Donor 2	Donor 3
TLR7	17.66	15.84	not expressed
TLR8	17.22	not expressed	not expressed
TLR3	18.04	not expressed	16.80
TLR9	14.00	10.78	13.79

Supplementary Figure 11: Influenza particles are not always found in the same platelets that express TLR7. a. Confocal images of platelets from 3 different donors stained with TLR7, influenza-A and LAMP1 antibodies. Platelets isolated from these healthy human donors were incubated with WSN/33 influenza strain as in Supplementary Figure 7. Cells were permeabilized and stained with TLR7-APC; influenza A-FITC, and the lysosomal marker LAMP1-DyLight405. **b.** Expression of all endosomal TLRs in isolated human platelets of the donors in (a), as assessed by qPCR. Expression was normalized to the housekeeping gene ACTB.

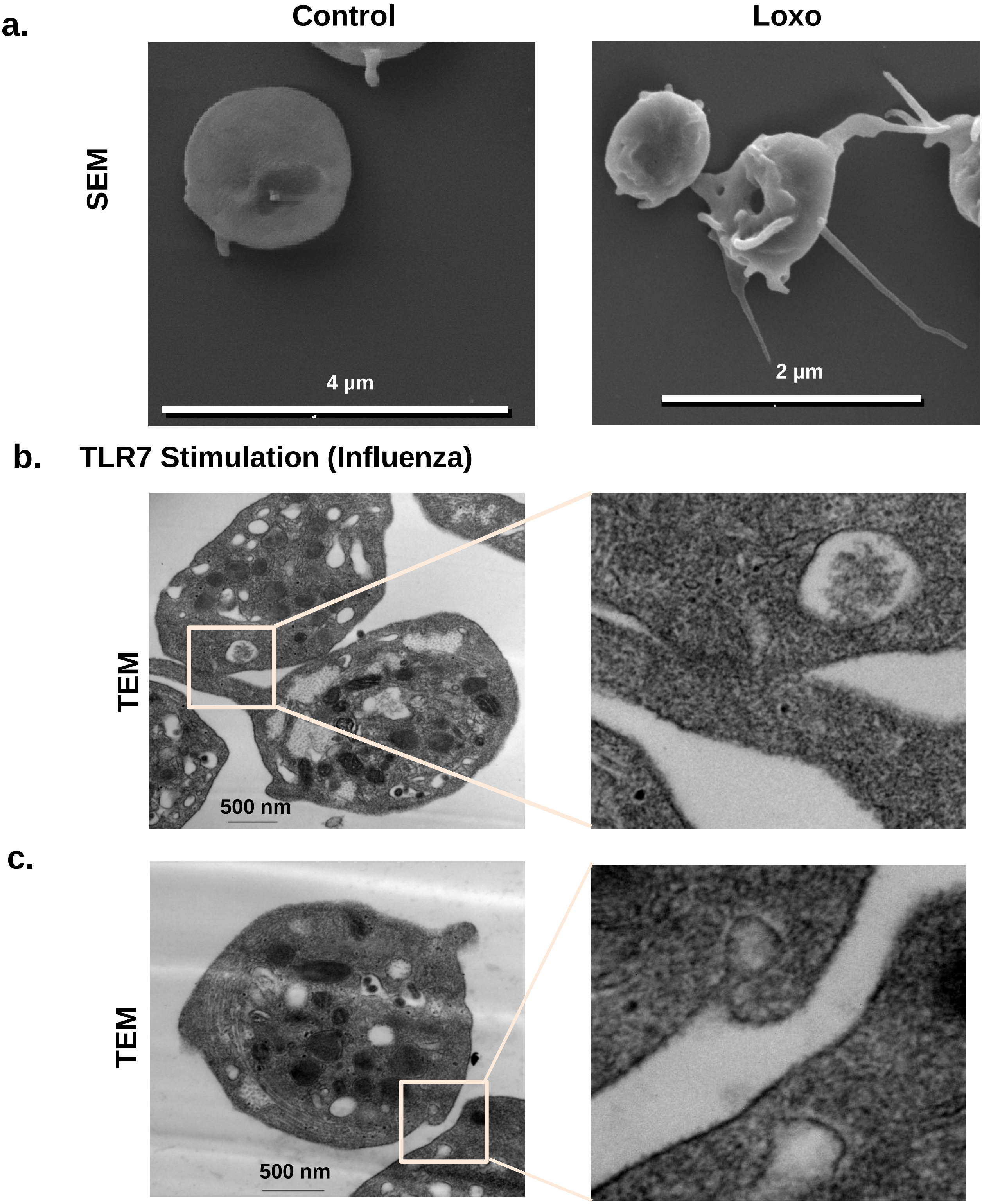

Supplementary Figure 12: TLR7 activation of platelets leads to platelet cross-communication. Isolated human platelets were TLR7-stimulated (with Loxo or influenza). **a.** Scanning electron micrographs (SEM) of platelets stimulated with Loxo for 15 min. **b-c.** Transmission electron micrographs (TEM) of platelets stimulated with WSN/33 for 30 min showing **b.** fusion of membrane between two platelets, **c.** clear separation and the cell wall of two platelets.

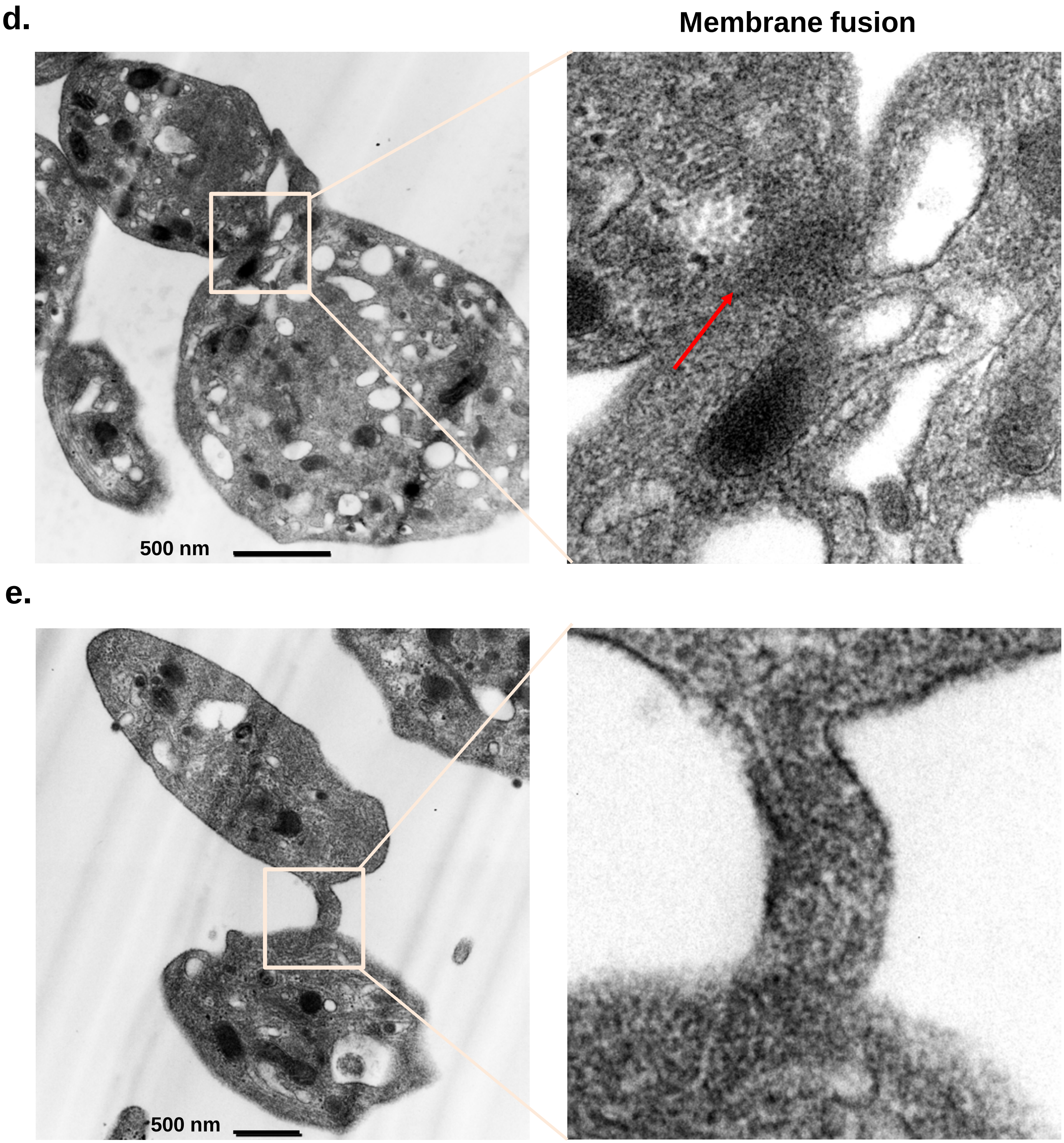

Supplementary Figure 12 (continued): TLR7 activation of platelets leads to platelet cross-communication. Isolated human platelets were TLR7-stimulated with influenza. **d-e.** Transmission electron micrographs (TEM) of platelets stimulated with WSN/33 for 30 min showing **d.** and **e.** additional images of neighboring platelets with fused membranes.

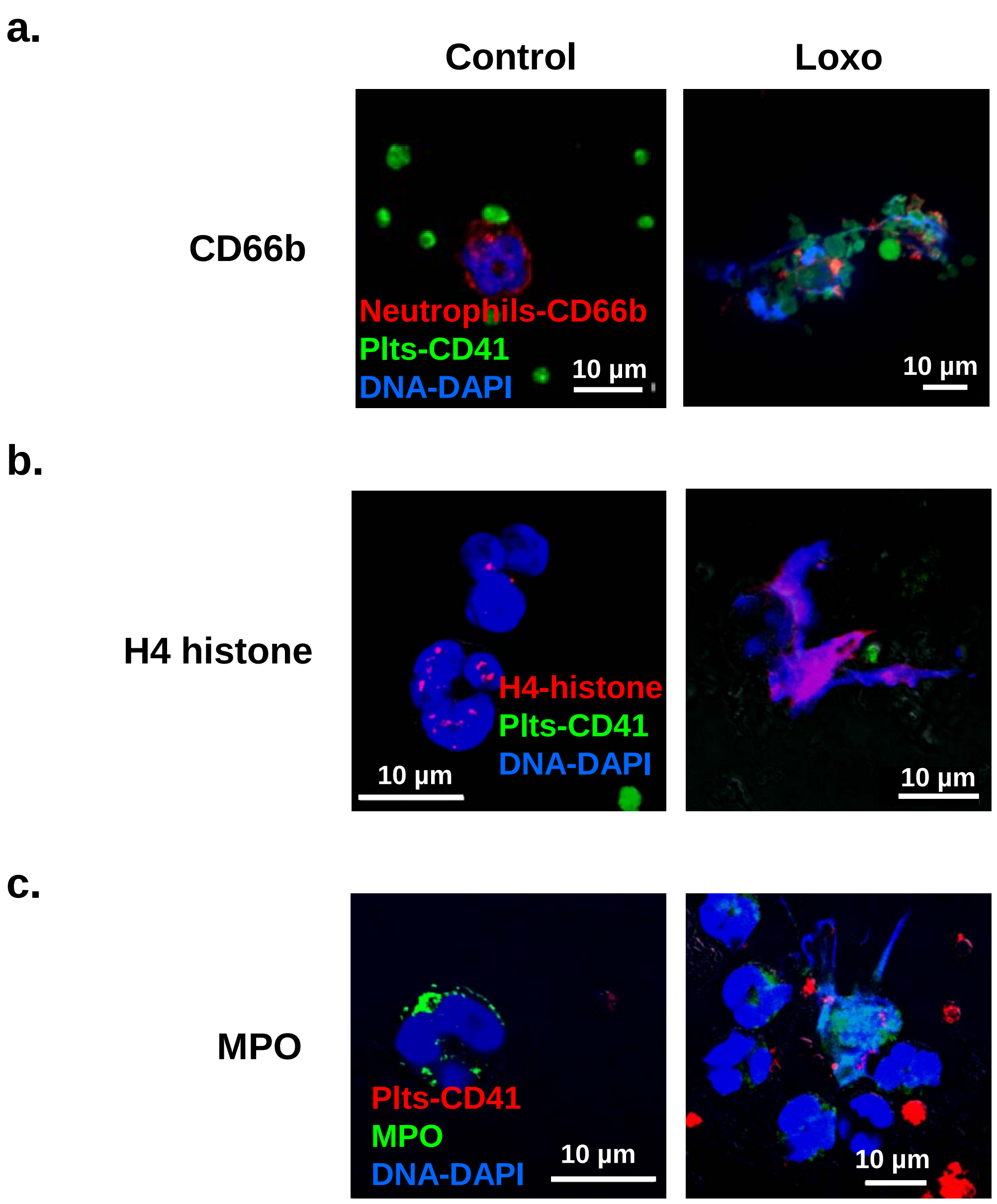

Supplementary Figure 13: Ex vivo treatment of human blood with a TLR7 agonist leads to DNA release from neutrophils. Intravenous blood was treated for 30 min with TLR7 agonist (Loxo, 1 mM) at 37°C and constant rotation of 1000 rpm. **a.** Blood was stained with CD66b, CD41 and DAPI after fixation. **b.** Blood was additionally stained with Histone H4. **c.** Blood was additionally stained with MPO. Panels are representative of confocal microscopy images taken from n=5 human donors. Antibody and dye staining are as follows: DAPI-DNA; CD41-platelets; CD66b-neutrophils. Abbreviations: Loxo- loxoribine, Plts-platelets, MPO-myeloperoxidase.

a.

Neutrophil isolation in this study

(from 20 mL of citrated blood)

				Patient Limits	
WBC	34.9	H	x10^9/L	4.5	10.5
RBC	0.04	*L	x10^6/uL	4.00	6.00
Hgb	0.2	*L	g/dL	11.0	18.0
Hct	1.3	*L	%	35.0	60.0
MCV	+++++		fL	80.0	99.9
MCH	43.1	*H	pg	27.0	31.0
MCHC	13.3	*L	g/dL	33.0	37.0
Plt	0.	*L	x10^3/uL	150.	450.

Neutrophil isolation using Ficoll-Hypaque gradient

(from 100 mL of citrated blood)

				Patient Limits	
WBC	96.6	H	x10^9/L	4.5	10.5
RBC	0.13	*L	x10^6/uL	4.00	6.00
Hgb	0.6	*L	g/dL	11.0	18.0
Hct	4.1	*L	%	35.0	60.0
MCV	+++++		fL	80.0	99.9
MCH	48.3	*H	pg	27.0	31.0
MCHC	15.3	*L	g/dL	33.0	37.0
Plt	313.	*	x10^3/uL	150.	450.

b.

Neutrophil purity

(from 20 mL of citrated blood)

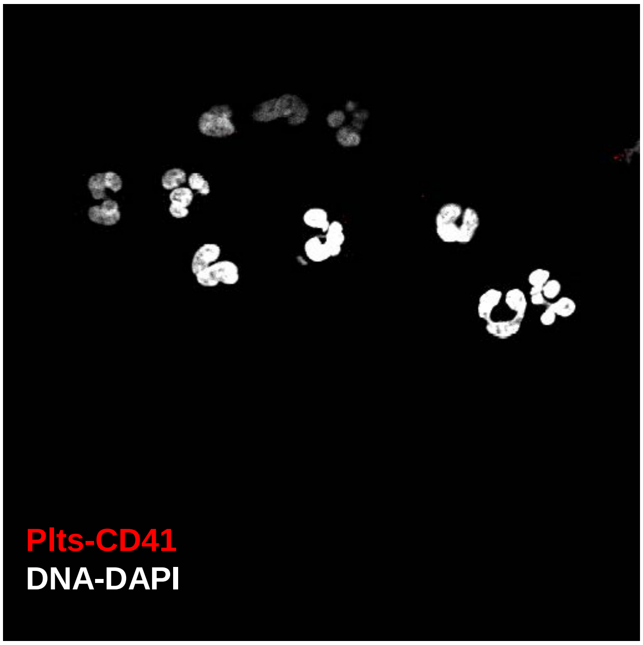

Supplementary Figure 14: Purity of neutrophils utilized throughout our study. a. Neutrophils were isolated using Cell Preparation Tubes (CPT) or Ficoll as described in the Methods. The platelet negative population was assessed by human blood counter analyzer (Ac.T 8). **b.** Confocal microscopy (DNA-white; platelets-red [CD41]) confirmed the absence of mononuclear cells in the neutrophil preparation.

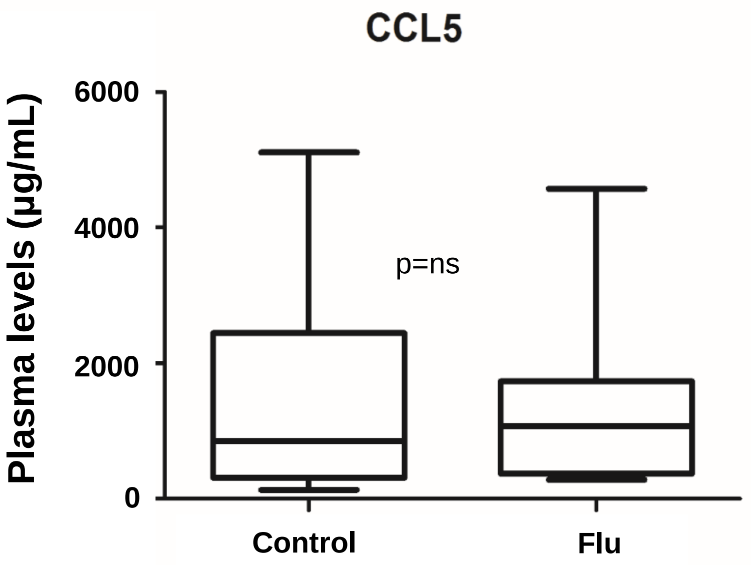

Supplementary Figure 15: Levels of CCL5 (RANTES) in plasma of influenza-infected patients. CCL5 was measured in the plasma of healthy donors (n=14) and influenza-infected patients (n=18). The graph represents average ± SD; significance was assessed by Mann-Whitney U test, p=0.8942.

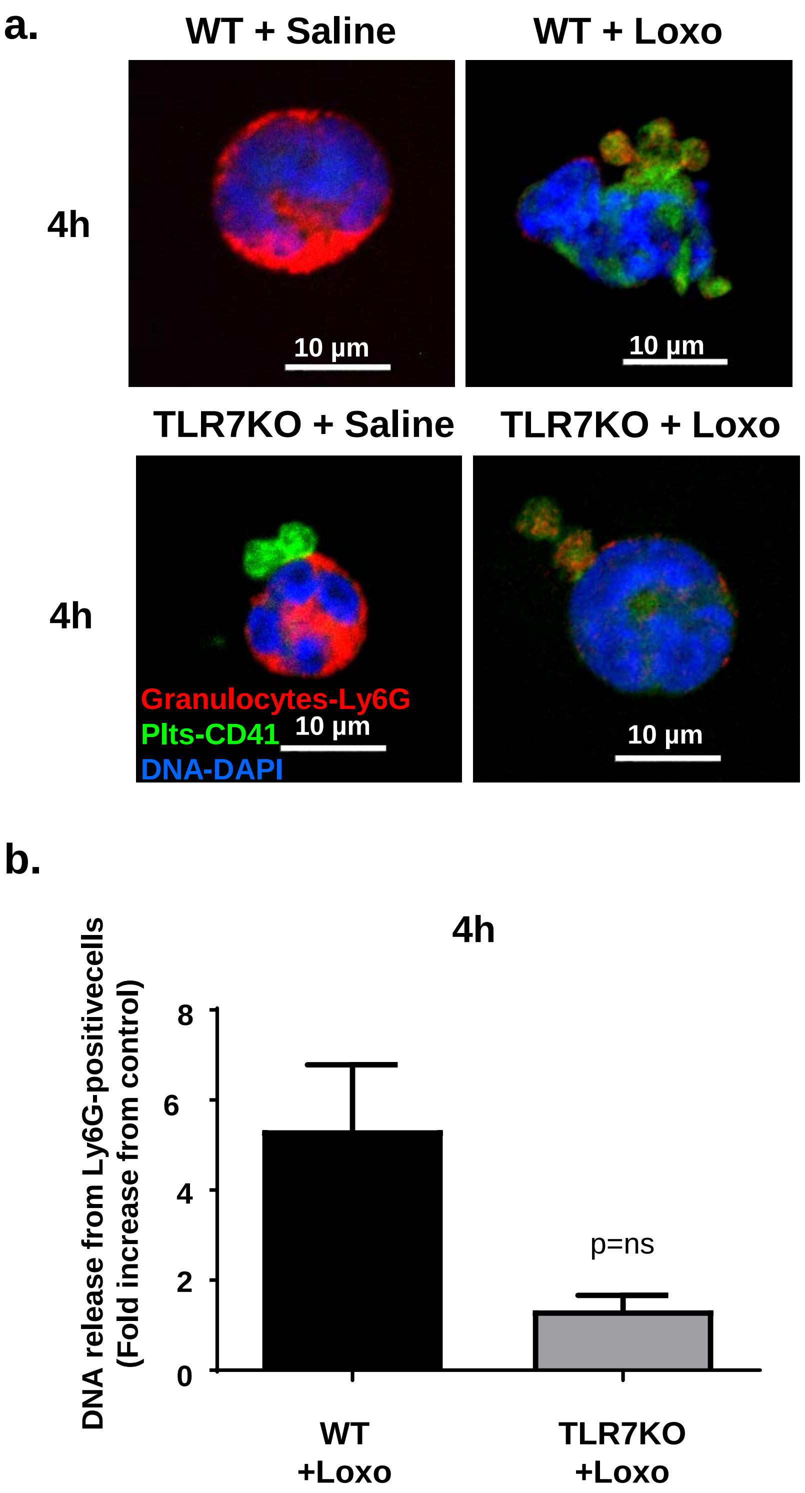

Supplementary Figure 16: Neutrophil-DNA release in mouse blood after 4 h of in vivo TLR7 stimulation. WT and TLR7 KO mice were injected intraperitoneally with TLR7 agonist (Loxo). Blood was collected by cardiac puncture at 4 h and immediately fixed (red blood cells were lysed at the same time). **a.** Representative images of neutrophil-DNA release (at 4 h post-Loxo stimulation) resolved by confocal microscopy. **b.** Quantitation of released neutrophil-DNA in blood of mice (n=4/group) at 4 h after agonist stimulation. Graph represents the average ± SD, p=0.06; assessed by an unpaired t-test (2-tail value); no statistical significance was observed at 4 h.

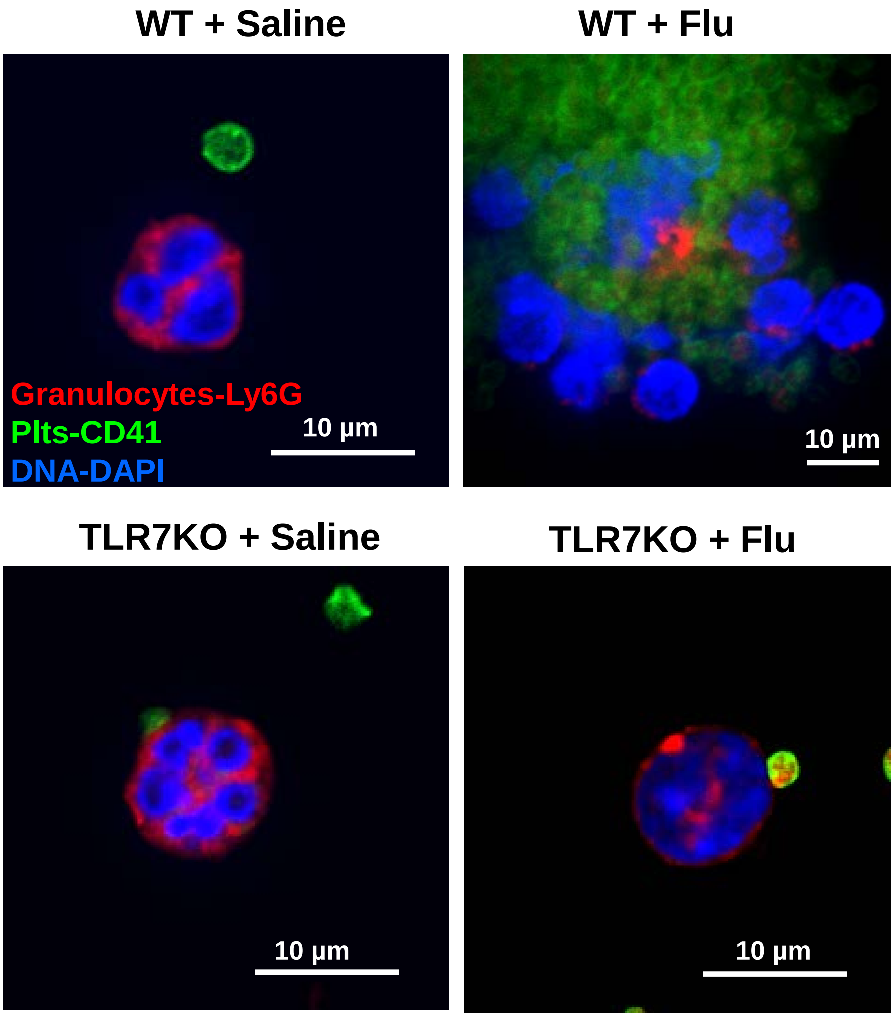

Supplementary Figure 17: Neutrophil-DNA release in murine blood after 24 h of in vivo infection with influenza. WT and TLR7 KO mice were intranasally infected with influenza (PR8 strain, 40,000 pfu in 30 µL). Blood was collected by cardiac puncture at 24 h and immediately fixed (red blood cells were lysed at the same time). Representative images of neutrophil-DNA release (at 24 h post influenza infection) resolved by confocal microscopy and stained with neutrophil marker Ly6G in addition to platelets (CD41) and DNA (DAPI). Representative images of n=4 mice. Quantitation of DNA release is included in Figure 9d.

a.

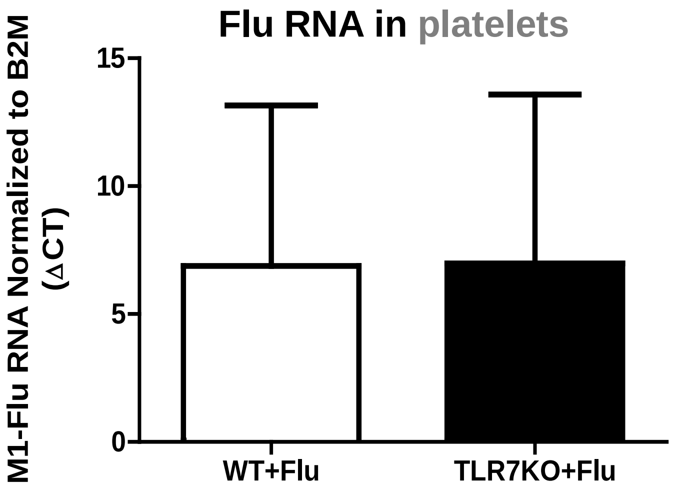

Flu RNA in plasma

WT+Flu	TLR7KO+Flu
n=8	n=8
1 (Ct=29.8)	3 (Ct=29.4±3)

b.

WT + Saline **WT + Flu**

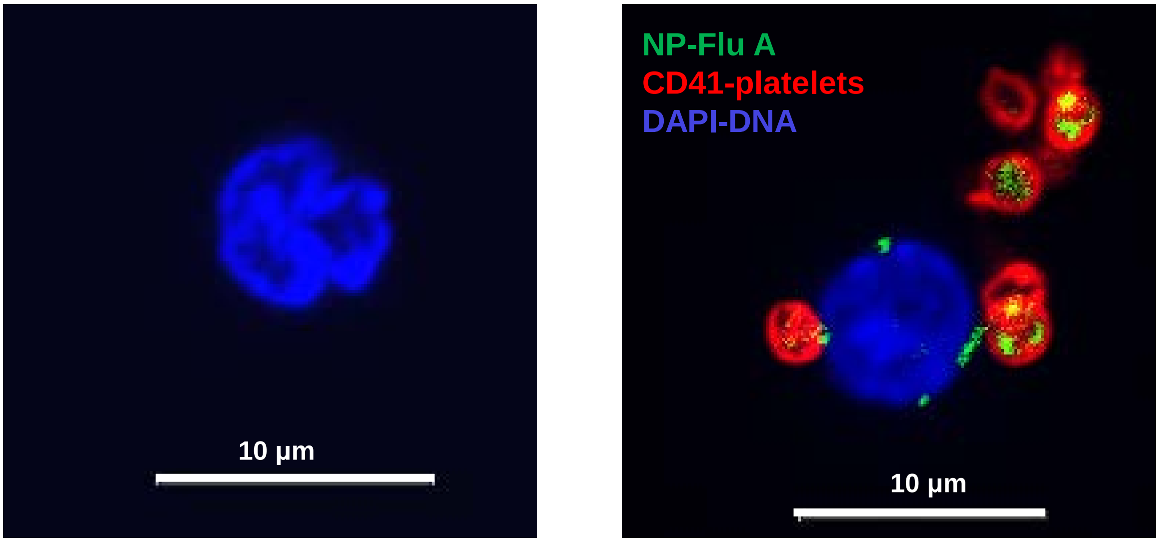

Supplementary Figure 18: Presence of influenza in murine blood post-infection. Mice were infected with influenza A strain PR8 as described in the Methods for 24 h. **a.** Influenza RNA levels (assessed by primers for the M1 gene) in isolated murine platelets and plasma at 24 h post-infection as assessed by qPCR and reported as fold change from the housekeeping gene Beta-2-Microglobulin (B2M). n (platelets)=5/mice per group. Graph represents the average ± SD. **b.** Confocal microscopy of murine blood stained with antibodies for the nuclear protein of influenza A shows the presence of influenza in platelets and neutrophils associated with platelets. Representative images of n=5 mice.

Supplementary Table 1. Characteristics of the Influenza Study Patients (N=18) and Controls (N=15).

**Characteristics**	**Controls (n=15)**	**Flu (n=18)**
**Age (years)**	41±17	65±18
**Women, n (%)**	9 (60%)	9 (50%)
**Influenza A, n (%)**	n/a	15 (83%)
**Aspirin, n (%)**	0	8 (44%)
**WBC ($x10^9$/L)**	4.7 ± 1	6.3 ± 2
**Platelets ($x10^3$/μl)**	229 ± 54	184 ± 62
**RBC ($x10^6$/μl)**	4.5 ± 0.9	4.2 ± 0.6

Supplementary Table 2. Influenza detection by qPCR (M1 in flu) or antibody staining (influenza A or B nuclear protein) in patients who were diagnosed with flu by nasal swab

Patient #	Sex	Influenza	Year of collection	Platelets Copy#/µg RNA	Whole blood Copy#/µg RNA	Antibody staining (NP)
1	F	A	2016	0.001	--	+
2	M	A	2016	0.001	--	+
3	F	A	2016	--	--	n.t.
4	F	A	2016	--	--	n.t.
5	M	A	2016	1.875	1.429	+
6	M	A	2016	8.300	--	+
7	M	A	2016	--	--	+
8	F	A	2016	--	--	n.t.
9	M	A	2017	--	--	+
10	F	A	2017	--	--	n.t.
11	M	B	2017	--	--	n.t.
12	M	A	2018	--	--	n.t.
13	M	A	2018	--	--	+
14	M	A	2018	--	--	n.t.
15	F	A	2018	--	--	+
16	F	B	2018	--	--	+
17	F	A	2018	--	--	n.t.
18	F	B	2018	--	--	+

*Influenza RNA was detected in 100 µL of whole blood. Antibody staining for influenza was done in 50 µL of whole blood (lysed and fixed before staining), or in isolated platelets; samples were randomly selected. Blood (or platelets) from healthy donors was negative for influenza either by qPCR (n=10) or antibody staining (n=5). n.t.=not tested.

Supplementary Table 3. TaqMan Gene Expression Assays Used in this study

RNA	Catalog Number	Assay Name	Custom Sequence
**Influenza A**	4331348	FLUA_M1_F	AAGACCAATCCTGTCACCTCTGA
		FLUA_M1_R	CAAAGCGTCTACGCTGCAGTCC
		FLUA_M1_P	TTTGTGTTCACGCTCACCGT
**Influenza B**	4331348	FLUB_HA-188_F	AGACCAGAGGGAAACTATGCCC
		FLUB_HA-F347_R	CTGTCGTGCATTATAGGAAAGCAC
		FLUB_HA-273_P	ACCTTCGGCAAAAGCTTCAATACTCCA
**TLR7**	4331182	Hs01933259_s1	N/A
**TLR8**	4331182	Hs00152972_m1	N/A
**TLR3**	4331182	Hs01551078_m1	N/A
**TLR9**	4331182	Hs00370913_s1	N/A

*Influenza Taqman primers used in this study were obtained from the World Health Organization website (http://www.who.int/influenza/gisrs_laboratory/molecular_diagnosis/en/)

Supplementary Note:

Previous studies have reported susceptibility to hepatitis C infection as a function of TLR7 or TLR8 polymorphism [1]. We aimed to analyze the published polymorphism sites (rs179009; rs3764879) utilizing available data from the FHS (Offspring Cohort, Visit 8). There are no complement C3 measurements available from the plasma of this cohort; however, there are complement C2 measurements, so we analyzed the above-mentioned polymorphisms with the protein quantitative trait locus (pQTL) of C2 in blood cells. C2 is located upstream of the C3 component of the complement system and is necessary for the processing of C3. There were no pQTLs of these two SNPs for C2 at P<1e-6 in this cohort. Future studies, beyond the scope of this one, are necessary to address polymorphism in TLR7 and severity of influenza infection.

Supplementary References:

1 Weyrich, A. S. & Zimmerman, G. A. Platelets in lung biology. *Annu Rev Physiol* **75**, 569-591, doi:10.1146/annurev-physiol-030212-183752 (2013).
