# Supplementary material for: The role of platelets in mediating a response to human influenza infection
Source: Nat Commun. 2019 Apr 16;10:1780. doi: 10.1038/s41467-019-09607-x (PMC6467905; doi:10.1038/s41467-019-09607-x)
Supplement: Supplementary file 3 — Reporting Summary [file 41467_2019_9607_MOESM3_ESM.pdf]

nature research

Corresponding author(s): DBPR NCOMMS-18-13908C

Last updated by author(s): Mar 8, 2019

Reporting Summary

Nature Research wishes to improve the reproducibility of the work that we publish. This form provides structure for consistency and transparency in reporting. For further information on Nature Research policies, see Authors & Referees and the Editorial Policy Checklist.

Statistics

For all statistical analyses, confirm that the following items are present in the figure legend, table legend, main text, or Methods section.

n/a	Confirmed	
☐	☒	The exact sample size (*n*) for each experimental group/condition, given as a discrete number and unit of measurement
☐	☒	A statement on whether measurements were taken from distinct samples or whether the same sample was measured repeatedly
☐	☒	The statistical test(s) used AND whether they are one- or two-sided<br>*Only common tests should be described solely by name; describe more complex techniques in the Methods section.*
☒	☐	A description of all covariates tested
☒	☐	A description of any assumptions or corrections, such as tests of normality and adjustment for multiple comparisons
☐	☒	A full description of the statistical parameters including central tendency (e.g. means) or other basic estimates (e.g. regression coefficient) AND variation (e.g. standard deviation) or associated estimates of uncertainty (e.g. confidence intervals)
☐	☒	For null hypothesis testing, the test statistic (e.g. *F*, *t*, *r*) with confidence intervals, effect sizes, degrees of freedom and *P* value noted<br>*Give P values as exact values whenever suitable.*
☒	☐	For Bayesian analysis, information on the choice of priors and Markov chain Monte Carlo settings
☒	☐	For hierarchical and complex designs, identification of the appropriate level for tests and full reporting of outcomes
☒	☐	Estimates of effect sizes (e.g. Cohen's *d*, Pearson's *r*), indicating how they were calculated

Our web collection on statistics for biologists contains articles on many of the points above.

Software and code

Policy information about availability of computer code

Data collection	No software was used besides excel
Data analysis	GraphPad Prism 5 or 7

For manuscripts utilizing custom algorithms or software that are central to the research but not yet described in published literature, software must be made available to editors/reviewers. We strongly encourage code deposition in a community repository (e.g. GitHub). See the Nature Research guidelines for submitting code & software for further information.

Data

Policy information about availability of data

All manuscripts must include a data availability statement. This statement should provide the following information, where applicable:

- Accession codes, unique identifiers, or web links for publicly available datasets
- A list of figures that have associated raw data
- A description of any restrictions on data availability

Data Availability statement is included after the Method section

Field-specific reporting

Please select the one below that is the best fit for your research. If you are not sure, read the appropriate sections before making your selection.

☒ Life sciences ☐ Behavioural & social sciences ☐ Ecological, evolutionary & environmental sciences

For a reference copy of the document with all sections, see nature.com/documents/nr-reporting-summary-flat.pdf

Life sciences study design

All studies must disclose on these points even when the disclosure is negative.

Sample size	Based on previous viral studies that we have done 4-5 mice per group assuming that at least 80% survival.
Data exclusions	There were no mice that were excluded, unless they didn't survive to the time point of interest. For the human samples there were no exclusions except when there was not sufficient reproducibility (only one sample)
Replication	We have run all human a plasma samples in duplicates when it comes to ELISAs. Throughout the manuscript, we included not less than 3 different biological representations throughout the manuscript. Any other limitations are explained in the manuscript.
Randomization	There is no method of randomization in the human samples. Animal randomization in the anti-platelet experiment is described on pg. 25.
Blinding	There was no blinding used in this study

Reporting for specific materials, systems and methods

We require information from authors about some types of materials, experimental systems and methods used in many studies. Here, indicate whether each material, system or method listed is relevant to your study. If you are not sure if a list item applies to your research, read the appropriate section before selecting a response.

Materials & experimental systems

n/a	Involved in the study	
☐	☒	Antibodies
☒	☐	Eukaryotic cell lines
☒	☐	Palaeontology
☐	☒	Animals and other organisms
☐	☒	Human research participants
☒	☐	Clinical data

Methods

n/a	Involved in the study	
☒	☐	ChIP-seq
☒	☐	Flow cytometry
☒	☐	MRI-based neuroimaging

Antibodies

Antibodies used	anti-human: CD41-FITC or CD41-APC (clone HIP8, eBioscience, CA, USA, cat# 11-0419 and cat# 17-0419), CD66b-APC (clone G10F5, eBioscience, cat# 17-0666), MPO-FITC (clone MPO455-8E6, eBioscience, cat# 11-1299), Histone H4-AF647 (clone 31830, Abcam, MA, USA, cat # ab197515, also recognizes mouse), Histone H3 (Abcam, cat# ab1791, followed by FITC-conjugated Goat Anti-Rabbit IgG H&L secondary antibody (Abcam, cat #ab6717); TLR7-APC (clone 4G6, Novus Biologicals, cat# NBP2-25274APC), LAMP-1/CD107a -DyLight 405 (clone 5E7, Novus Biologicals, cat# NBP2-52721V), CD63-BV421 (clone H5C6, Biolegend, cat #353029); anti-mouse: CD41-FITC (clone MWReg30, eBioscience, cat# 11-0411), Ly6G-APC (clone RB6-8C5, eBioscience, cat# 17-5931); and Influenza A-NP-FITC (Abcam, cat# ab20921), Influenza B-NP-FITC (Invitrogen, cat# MA1-7306).
Validation	To the best of our knowledge all antibodies used here are validated by manufacturer and used in previously published studies. Influenza A and B antibodies showed presence only in the infected samples as shown in our figures.

Animals and other organisms

Policy information about studies involving animals; ARRIVE guidelines recommended for reporting animal research

Laboratory animals	TLR7 KO mice were originally obtained from S. Akira and then backcrossed to C57BL/6J (WT) for at least 10 generations (32). C57BL/6J mice were purchased from the Jackson Laboratory (ME, USA, cat# 000664). These studies used sex- and (12-16 weeks) age-matched mice.
Wild animals	We have not used any wild animals for this study.
Field-collected samples	We have not used any field-collected animals for this study.
Ethics oversight	The University of MA Medical School IACUC Committee

Note that full information on the approval of the study protocol must also be provided in the manuscript.

Human research participants

Policy information about studies involving human research participants

Population characteristics	All relevant information concerning the population used in this study is included in Supplemental Table 1
Recruitment	Patients age above 21 that presented with verified influenza swab were included according to their agreement to participate.
Ethics oversight	The University of MA Medical School IRB Committee

Note that full information on the approval of the study protocol must also be provided in the manuscript.
